# An Essay on Artificial Teeth, Obturators, and Palates, with the Principles for Their Construction and Application; Illustrated by Twenty-Six Cases and Twenty-One Plates

**Published:** 1840

**Authors:** 


### BIBLIOGRAPHICAL.

*An Essay on Artificial Teeth, Obturators, and Palates, with the principles for their construction and application; illustrated by twenty-six cases and twenty-one plates. By* LEONARD KOECKER, *Surgeon Dentist, Doctor in Medicine and Surgery, &c. London—S. Highley,* 32 *Fleet st., p p.* 194.

The above is a title of a work, which, though it was published five years ago, few copies of it have found their way to this country. Presuming, therefore, that it is but little known to our professional brethren on this side of the water, a brief notice of it may not be unacceptable to them. That it is a work of merit, the author's name is a sufficient guarantee. Dr. Koecker has been long known to the profession both in Europe and America as an author and practioner, and to his pen the literature of Dental Surgery is indebted for some of its richest and most valuable contributions. Having devoted himself to the profession, with the most untiring energy and zeal, for upwards of thirty years, and being possessed of a bold, active and enquiring mind, he has acquired a degree of skill in the treatment of dental and maxillary diseases possessed by, comparatively, very few. His opinions, therefore, are entitled to the highest consideration; and although we do not concur with him in all which he has advanced, we would not, on that account, withhold from him that high meed of praise to which we conceive him to be justly and richly entitled. As a dental pathologist and practitioner, he ranks among the very first in Europe. His "Principles of Dental Surgery," evince extensive practical observation; they are based upon a correct knowledge of the laws of disease, and if energetically followed out, cannot be otherwise than successful.

The first chapter of the work before us, treats "on the use and abuse of artificial teeth," and after speaking of the advantages that may result from these, when "*judiciously* and skilfully inserted," the author says—"the artificial may be rendered nearly an equivalent substitute for the natural organ. But as no mechanical part of any branch of surgery can be carried so near to perfection, so none damands *greater skill and nicety*, combined with a scientific knowledge in the practitioner. An injury done to the various parts of the mouth, by the insertion of one or more teeth upon erroneous principles, must render all future restorative efforts less useful; and it will often happen, that in correcting a small evil, a much greater will be created. Thus, one tooth may be inserted upon so false a principle, and in so unskilful a manner, as to loosen all around it, and eventually to cause the loss of the entire set.

The evil spoken of here is one of very common occurrence, and has been instrumental in bringing down upon the profession much obloquy and reproach. Abuses of this kind, however, must be expected, as long as the duties of the art are permitted to be exercised by every one who is disposed to assume them.

A little further on, Dr. K. makes the following judicious remarks—"A dentist who is competent to the discharge of his duties will not only supply the specific loss, or remedy the defect ; but he will detect and remove the latent evil. It is as much a part of his duty to see that the cause which has occasioned the particular loss or injury brought under his notice, shall not continue its destructive operation, as it is to supply the deficiency."

Indeed, the restoration of the natural teeth to health, and such other parts of the mouth as may be in a morbid condition, is of much greater consequence, than the insertion of artificial teeth. The first care, therefore, of the dentist should be directed to this object.

Again, the author pertinently observes—"It is to be regretted, that dental surgery, as a branch of the healing art, is yet a very obscure science ; and, while it is, at all events—not less intricate and abstruse than any other of its branches, it is by far the least understood by medical men, as well as by the public at large. It is also deprived of every protection by the medical or surgical faculty. It is principally from these causes that it has not acquired that importance to which its great difficulties and utility so justly entitle it, while it is overrun by those who degrade the very name of the profession, and endanger the health and comfort of the public."

"These circumstances are particularly productive of the great difficulty existing to the public, in drawing a just distinction between the ignorant and the skilful dentist ; and if these facts are duly considered, we can no longer be surprised at the frequent occurrence of individuals, even of the most exalted stations in life, so much deluded by mistaken views and false notions, as to apply to the uneducated practitioner, for the insertion of artificial teeth, at a period when the various diseases of their mouths require the most comprehensive treatment from the *surgeon dentist.*

He concludes the chapter, with a case illustrative of the pernicious effects that result from the employment of badly constructed artificial teeth and the advantages that are derived from them when properly applied. The subject of the case was 55 years of age, and had, from bad treatment of his teeth in early life, and in consequence of the injudicious insertion of a few artificial at this period,—neglecting, at the same time, the treatment necessary to the restoration and preservation of his others, lost, in the course of ten or fifteen years, all he had. His health, in the mean time, had become greatly impaired.

To remedy, as far as possible, the loss of his natural teeth, he had recourse to double sets of artificial; but these, in consequence of being badly contrived, he was unable to wear, with any satisfaction or advantage ; they having been so illy adapted, that his mouth was kept in a constant state of irritation by them.

Loosing confidence in his dentist, he at length applied to Dr. K. who furnished him with a set properly constructed and adapted, which not only enabled him to masticate with ease, but also restored to his face its original healthy contour, and was instrumental in restoring him his lost vigour and health.

The second chapter treats "*on the difficulties accompanying the insertion of artificial teeth,*" and here we find many judicious and correct observations, which, did our space permit, we should like to notice. For want of this, however, we pass on to a brief notice of the third chapter, which is devoted to the consideration of "*the surgical and mechanical principles, for the application of artificial teeth—their indications—the modes and materials best calculated for their preparation and insertion, and the means of their attachment.*"

After a few general introductory remarks, the author proceeds to describe the necessary surgical treatment of the mouth, preparatory to the insertion of artificial teeth, which consists in the removal of all decayed roots, dead teeth and extraneous matter of every kind, whereby the gums or any part of the mouth may be kept in a state of morbid excitement, and the restoration of such teeth as may be permitted to remain without injury to the contiguous parts. "*The indications and counter-indications, for the use of artificial teeth,*" are next considered, and here he says,—"A correct decicision for the application of artificial teeth, is frequently dependent on very deliberate consideration, which can be estimated only by a perfect knowledge, *founded* on considerable experience and good judgement, of all the changes and effects which are the result of time, age, and mastication, as well as of the diseases, *and the natural curative powers of the teeth,** and of the parts in any way related to them." Again he observes, "The use of one or more artificial teeth, is properly indicated, and must be useful and necessary, if they can be applied, without bearing too much upon the remaining teeth ; and if they can be rendered an assistance in mastication, pronunciation, or the improvement of appearance." Under other circumstances we are told that they are never indicated.

Speaking of the materials proper for artificial teeth, Doct. Koecker says, "the only kind of artificial teeth, and the materials for their preparation, that I have used, and use at present, are the teeth of the sea-horse

---

* Without for a moment questioning the vitality of the teeth, and that they are supplied with blood vessels, nerves, &c., facts that have long since been fully established, we cannot subscribe to the doctrine, that these organs are endowed with *restorative powers*, as is evidently implied by the expression,—"*natural curative powers of the teeth.*"

human teeth, and single mineral ro terro-metallic teeth, mounted in various ways upon no other metal than gold or platina.

As it regards the use of the sea-horse teeth for the construction of artificial, although recommended and used by many of our most distinguished practitioners, they are in our opinion exceedingly objectionable. Our reasons for opposing the use of this material for artificial teeth have been given at length in another place,* it is unnecessary, therefore, to recapitulate them.

Adverting to the manufacture of porcelain teeth in blocks, Dr. K. thus correctly remarks, "repeated attemps have been made in Paris, London and Philadelphia, to render such preparations more perfect; but success, I fear, must be considered hopeless, from the simple fact that it would be founded on principles contrary to the laws of chemistry; for such whole pieces being made of a soft paste, which is afterwards baked in the oven by a similar process to the manufacture of china, they are necessarily exposed to the changes in size and form which the heat produces."

From this it will be seen, that it is next to impossible, that a series of this description of teeth can be made to fit with exactness to the inequalities of the parts upon which they must rest.

This chapter is concluded with a few appropriate remarks "*on the principles for constructing, and the means for attaching artificial teeth.*"

The manner of preparing and inserting, by means of plate, single artificial teeth, constitutes the subject of the next, or fourth chapter, illustrated with cases and plates, as have been the preceding ones. Passing from this to the fifth chapter, the insertion of sets of two or more teeth are there treated of, interspersed, as before, with illustrative and explanatory cases and plates. In the chapter following, the consideration of sets "*embracing a considerable part or the whole of the upper jaw,*" engages the author's attention. Succeeding to this, is one descriptive of "*the principles for preparing and inserting double sets,*" but we cannot allow ourselves to pass thus hastily over these parts of the work without observing that Doctor Koecker evinces a most thorough and perfect knowledge of the several branches of the subject embraced within them.

The pivoting method of inserting artificial teeth next engages his attention; and here, I would observe, that while many of his views, in regard to the manner of their application and utility are correct, there are others that are evidently erroneous. For example—speaking of the insertion of artificial teeth by this method, he says it is "*always attended with more or less irritation.*" Now that this is very frequently the case, we do not deny—but that it is not *always*, hundreds of cases might be adduced to prove; but it is a method of insertion, I am free to admit which, as Dr. K. correctly observes "requires great caution. When properly done however, and upon a healthy root, I have no hesitation in saying that it is one of the best and most satisfactory methods that has ever been adopted.

---

*** See Dental Art, Practical Treatise on Dental Surgery.**

The principal objection, however, that he urges against this operation is, that the root of a tooth, often losing its lining membrane, ceases to possess vitality, and becomes obnoxious to the surrounding living parts—besides, that there is always a formation of matter in it or at its apex, and which, by the usual method of attaching the artificial crown, is prevented from escaping through the natural opening in the roots, and that this obstruction gives rise "to gum boils, or small fistulous abscesses."

The root of a tooth is supplied with blood vessels, nerves, &c., from its investing as well as its lining membrane, and often retains a sufficient degree of vitality, to prevent it from becoming hurtful or obnoxious to the socket for many years after the destruction of the latter ; consequently, the first objection will not apply to, at least, all cases ; and as it regards the accumulation of matter in the root, that may be prevented in a much better way than that proposed by Dr. Koecker, which consists in the insertions of the tooth, so that it may be from time to time removed by the patient for its escape. The necessity for this, is superceded by the formation of a groove on the side of the pivot, as recommended by Dr. L. S. Parmly, of New Orleans. A tooth inserted in this way will often remain from five to ten or fifteen years without producing any unpleasant effects whatever ; and to subserve too, all the purposes of a natural organ.

The concluding part of the work, describes the manner of forming and applying "*artificial obturators and palates*," which every dentist should understand.

In conclusion, we would remark, that the whole work is replete with instruction and valuable information on this part of the art of the dentist, and had the author dwelt a little more minutely on the various manipulations connected with the construction of the various descriptions of artificial teeth on which he has treated, its value would have been greatly enhanced, and as it is, we regard it as one of the best—if not the very best treatise extant on the subject in the English language. It is, in short, a work that should be in the hands of every one engaged in the practice of dental surgery. BALT. ED.

